# Clinicopathologic Features Predictive of Distant Metastasis in Patients Diagnosed With Invasive Breast Cancer

**DOI:** 10.1200/GO.20.00257

**Published:** 2020-09-04

**Authors:** Basim Ali, Fatima Mubarik, Nida Zahid, Abida K. Sattar

**Affiliations:** ^1^ Department of Biological and Biomedical Sciences, Aga Khan University, Karachi, Pakistan; and Baylor College of Medicine, Houston, TX; ^2^ Medical College, Aga Khan University, Karachi, Pakistan; ^3^ Department of Surgery, Aga Khan University, Karachi, Pakistan

## Abstract

**PURPOSE:**

National Comprehensive Cancer Network and European Society for Medical Oncology guidelines suggest screening for distant metastasis (M1) in symptomatic patients or those with locally advanced breast cancer. These guidelines are based on studies that often used pathologic staging for analysis. Physician variability in screening for M1 has also resulted in overuse of diagnostic tests. We sought to identify clinicopathologic features at diagnosis that could guide testing for metastatic disease.

**METHODS:**

Patients diagnosed with invasive breast cancer between January 2014 and December 2015 were identified from our institutional database. Demographic and clinical variables were collected, including receptor profiles and clinical TNM staging. Rates of upstaging for each clinical stage and rates of concordance of pathologic and clinical staging were analyzed. Univariate analysis and multivariate regression analysis (*P* < .05) identified predictors of upstaging to stage IV disease.

**RESULTS:**

A total of 370 patients met the inclusion criteria. Seventy patients (18.9%) had metastatic disease at diagnosis. The rate of upstaging for stages I, IIA, IIB, and III were 0%, 5.6%, 18.8%, and 36.6%, respectively. Advancing clinical stage, tumor size, and nodal status resulted in a significantly higher rate (*P* < .001) of upstaging to M1 disease. Age and hormone receptor status were not associated with upstaging to stage IV disease. Clinical stages I-III were concordant with pathologic staging in 65(42.8%) of 152 patients (kappa’s index, 0.197; *P* < .000).

**CONCLUSION:**

Advancing clinical stage, tumor size, and nodal status at diagnosis were predictive of upstaging to M1 disease in patients with breast cancer. Distant metastatic workup should be considered in patients with clinical stage IIB disease or higher.

## INTRODUCTION

Breast cancer is the most common malignancy affecting women worldwide.^1^ The diagnosis of breast cancer warrants accurate staging to make therapeutic decisions and determine prognosis. However, recent studies show that inappropriate screening tests have contributed to increasing health care costs.^2-4^

**CONTEXT**

**Key Objective**
What are the clinicopathologic features that predict distant metastasis at diagnosis of breast cancer? Our study examined the clinical and pathologic features available at the time of diagnosis that will allow physicians to evaluate the need for metastatic workup, especially in a resource-limited setting.
**Knowledge Generated**
Only advancing tumor size and nodal status consistently predicted risk of distant metastasis at diagnosis in breast cancer. Metastatic workup should be considered for patients with clinical stage IIB disease or higher.
**Relevance**
Our findings show that routine metastatic workup may be safely omitted in early-stage breast cancer.

The National Comprehensive Cancer Network and European Society for Medical Oncology have issued guidelines recommending staging imaging to rule out distant metastasis only in symptomatic patients or in those with locally advanced (stage III) breast cancer.^5,6^ Many of the studies used to inform these guidelines have used pathologic staging for their analysis.^7,8^ Only the clinical and not the pathologic stage is available to the clinician at the time of initial presentation when the decision for obtaining metastatic workup is made. The pathologic stage after surgery can frequently be discordant with the clinical stage.^9,10^

Despite existing guidelines that discourage indiscriminate and routine use of metastatic workup, physician variability in obtaining such tests has been reported.^11,12^ There is often overuse of diagnostic tests, with unnecessary exposure to radiation and false-positive results that warrant additional workup, leading to delays in care and a burden on the health system with increasing health care costs. Conventional diagnostic tests for excluding metastasis include various combinations of chest x-ray; liver, abdominal, or pelvic ultrasound; bone scintigraphy; computed tomography (CT) of the chest, abdomen, and pelvis; and more recently, positron emission tomography (PET)–CT. The additional cost of these tests may also be a deterrent to seeking definitive care, thus resulting in delayed presentation with advanced disease.^13,14^ The aim of this study was to determine the clinicopathologic features of invasive breast cancer that are predictive of upstaging to stage IV and can aid physicians in their decision making for obtaining metastatic workup in patients with clinical stage I-III disease.

## METHODS

After obtaining approval from the Ethics Review Committee of Aga Khan University in Karachi, Pakistan, a retrospective review of a prospectively maintained institutional database of patients diagnosed with breast cancer was performed. All patients with invasive breast cancer diagnosed between January 1, 2014, and December 31, 2015, were included. Those with incomplete/irretrievable records, bilateral breast cancer, a second primary cancer, ductal carcinoma in situ, and recurrent and male breast cancer were excluded from the analysis.

Clinical parameters collected included patient age, clinical tumor size, and nodal status on physical examination and breast imaging (mammogram and ultrasound). The final clinical tumor size (cT) and clinical nodal status (cN) used for correlation with upstaging was the largest/highest of the estimated clinical tumor size (cT) and clinical burden of nodal disease (cN), whether on physical examination or breast imaging. At the time these patients were diagnosed, it was not our institutional policy to routinely perform histologic confirmation of axillary lymph nodes suggestive of metastasis. Most, but not all, patients underwent biopsy confirmation of axillary disease before undergoing surgery. For consistency, we sought to use clinical suspicion alone (based on examination and axillary imaging) to stage the axilla.

At our institution, metastatic workup is routinely performed in all patients with invasive breast cancer per patients’ requests (self-paying) or physician preference. The preferred imaging modalities to screen for distant metastasis are bone scintigraphy and CT scan of the chest, abdomen, and pelvis. However, to make the workup more affordable, a chest x-ray, liver ultrasound scan, or CT scan of the chest with liver cuts is often used. PET-CT was not available at our institution during the study period. Information on the imaging studies obtained for metastatic workup and their respective findings (ie, presence or absence of metastasis) was also recorded. Patients were determined to have stage IV disease based on characteristic findings on metastatic workup and/or multidisciplinary tumor board consensus. A second imaging modality, such as magnetic resonance imaging (MRI) and ultrasound, as appropriate, was used when necessary to confirm distant metastasis.

The pathologic features included in the analysis for predicting upstaging included those that are available after a core-needle biopsy, before definitive surgery (ie, histologic grade using the Bloom-Richardson system and estrogen, progesterone, and human epidermal growth factor receptor 2 [HER2] receptor status).

Patients identified as having metastatic disease (M1) on staging workup obtained at diagnosis either did not have surgery upfront or surgery at all. Although these patients with M1 disease were used in the analysis for predicting upstaging, they were excluded from the analysis of correlating clinical and pathologic stages. Thus, the pathologic tumor size (pT) and pathologic nodal status (pN) were collected only for patients who had surgery upfront and where the correlation of cT and cN with pT and pN, respectively, could be meaningful. For the purpose of this study, all TNM staging was performed and recorded according to the 8th edition of the American Joint Committee on Cancer staging system.

Frequencies were calculated for categorical variables. The rate of upstaging to stage IV disease was calculated for each stage and was defined as the number of patients confirmed to have distant metastasis on metastatic workup, divided by the total number of patients tested. For each pathologic stage, the rate of underestimation or overestimation of clinical stage (discordance) was calculated and defined as the number of patients with clinical stage lower or higher than the final pathologic stage, divided by the total number of patients within that pathologic stage.

Analysis was performed using χ^2^ test and Fisher’s exact test, as appropriate. Cohen’s kappa was calculated when comparing concordance of clinical and pathologic data. A *P* value < .05 was considered significant. A univariate analysis followed by multivariate regression analysis (*P* < .05) was conducted for clinicopathologic features.

## RESULTS

A total of 466 patients presented with invasive breast cancer during the study period, of whom 370 met the inclusion criteria. Median age at diagnosis was 51 years (range, 18-95 years). Surgical pathology reports, for the purpose of correlating clinical T and N with pathologic T and N, were available for 152 (41%) of patients because the remaining patients either had surgery after receiving neoadjuvant systemic therapy or did not have surgery at all (stage IV disease; Table 1).

**TABLE 1:**
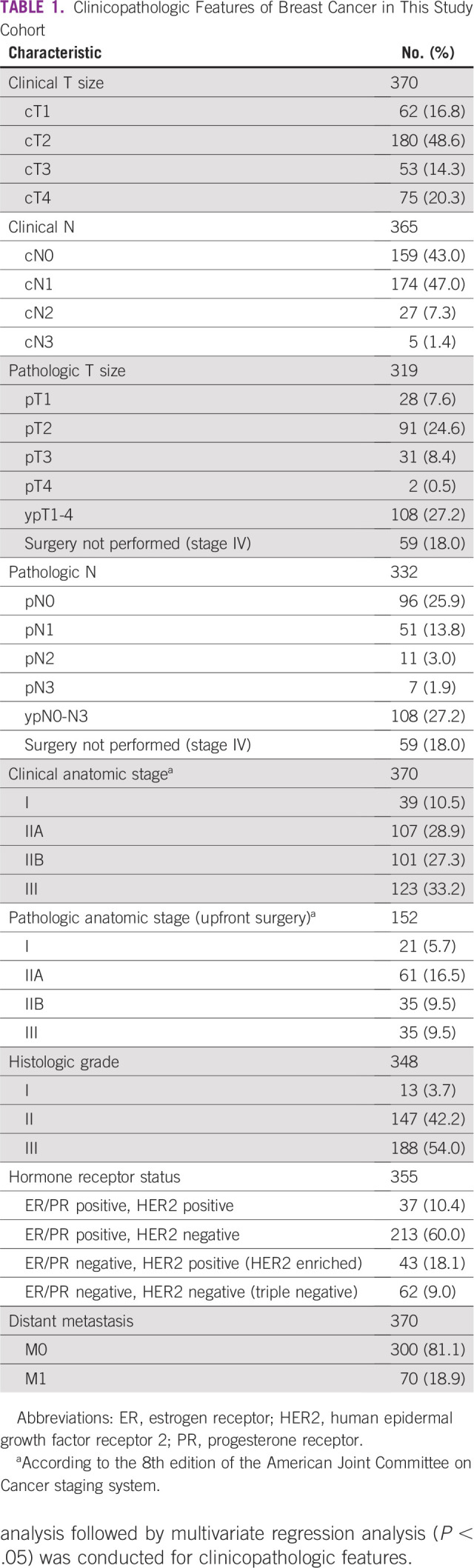
Clinicopathologic Features of Breast Cancer in This Study Cohort

Bone scans were performed in 336 (90.8%) of patients. The second most commonly performed study was CT of the chest with liver cuts, which was performed in 282 (76.2%) of patients. Other imaging techniques frequently used at our institution to screen for distant metastasis included chest x-ray (33.0%), CT of the abdomen and pelvis (45.1%), and ultrasound of the liver (20.0%). Thirty-eight (10.3%) of patients required an additional study, such as MRI of the spine or thyroid ultrasound to evaluate lesions considered suggestive of metastasis on other imaging studies.

A third of the patients (123 of 370) presented with locally advanced, clinical stage III disease. Overall, 70 (18.9%) of the patients were found to have distant metastasis on metastatic workup. Bone (n = 42, 11.4%) was the most frequently involved site, followed by lung (n = 30, 8.1%), and liver (n = 22, 5.9%). Brain metastasis was found in 4 (1.1%) of patients.

**FIG 1:**
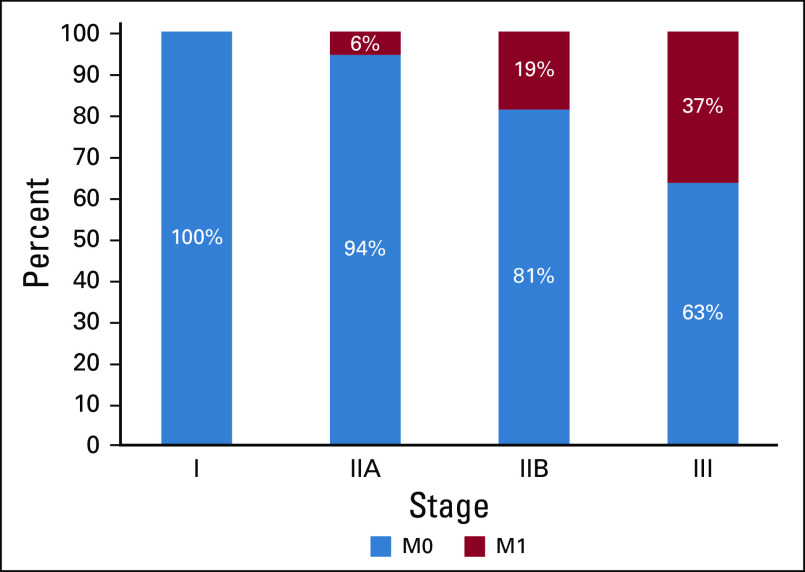
Patients with clinical stage IIB or III were most frequently upstaged to M1 after metastatic workup was obtained.

The rate of upstaging to stage IV (M1) significantly correlated with clinical anatomic stage (*P* < .001), clinical T size (*P* < .001), clinical N (*P* < .001), and histologic grade (*P* = .018; Table 2). Patients with clinical stages IIB (n = 19 of 101, 18.8%) and III (n = 45 of 123, 36.6%) were most frequently upstaged to M1 after metastatic workup was obtained (Fig 1). On multivariate analysis, the only clinicopathologic features predictive of metastatic disease were clinical T (*P* = .022) and N (*P* < .001) stage. Age, hormone, or HER2 receptor status were not found to be significant predictors of distant metastasis.

**TABLE 2:**
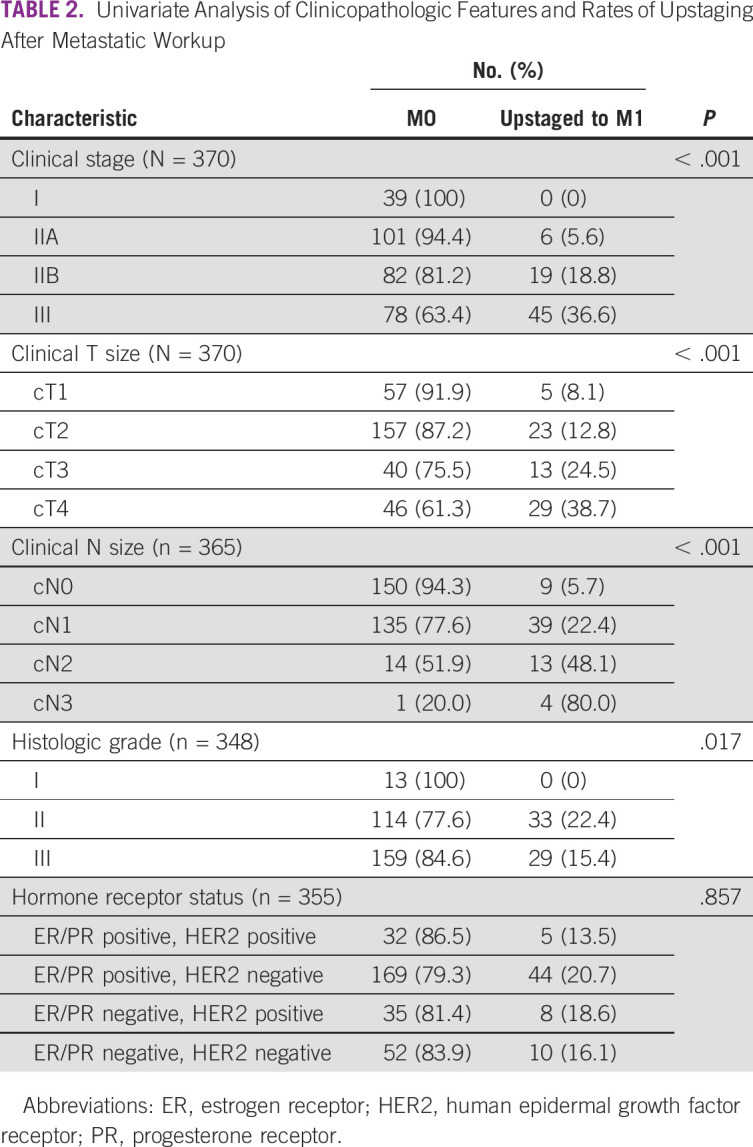
Univariate Analysis of Clinicopathologic Features and Rates of Upstaging After Metastatic Workup

**FIG 2:**
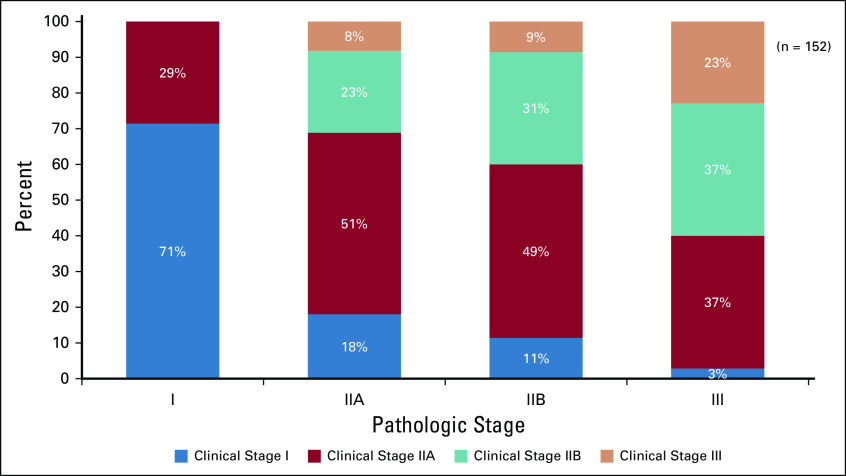
Pathologic stage in patients who received upfront surgery and the corresponding clinical TNM stages.

Overall, clinical stages I through III were found to be concordant with pathologic staging in fewer than half (n = 65 of 152, 42.8%) of the patients (kappa’s index 0.197; *P* < .000). In patients in whom there was discordance between clinical and pathologic stages (n = 87 of 152, 57.2%), the clinical stage was underestimated in 38.8% (n = 59 of 152) and overestimated in 18.4% (n = 28 of 152) of patients. The rates of underestimated clinical stage for pathologic stages IIA, IIB, and III were as follows: stage IIA: 18.0% (n = 11 of 61); stage IIB: 60% (n = 21 of 35); and stage III: 77.1% (n = 27 of 35; Fig 2)

## DISCUSSION

The prevalence of distant metastasis in the absence of clinical symptoms in patients with invasive breast cancer is reported to be between 1.2% and 6.4%.^3,8,15^ This is also reflected in national and international guidelines recommending against routine use of metastatic workup in patients with early-stage disease (stages I-IIB) or asymptomatic patients.^5,6^ Many of the guidelines have been based on studies that used pathologic staging to analyze rates of clinical upstaging to stage IV disease. Path-ologic stage is frequently discordant with the clinical stage, with studies reporting discordance in 32%-40% of patients.^9,10^

Our study is unique for several reasons. To our knowledge, this is the first study to report upstaging rates for each clinical stage in a Pakistani population. We also attempted to identify clinicopathologic features available at the time of diagnosis to predict upstaging to stage IV disease and thus inform subsequent decisions for obtaining metastatic workup. In addition, we divided stage II patients into stage IIA and stage IIB to demonstrate a greater risk of distant metastasis in the latter group. Finally, we correlated our clinical and pathologic staging to estimate concordance between both and to understand the impact of extrapolating data from pathologic staging to guide decisions made using clinical staging.

In a study of 412 patients, Puglisi et al^7^ found that 5.6% of patients with stage II disease had bone metastasis at presentation using pathologic staging. Another large study of 2,612 patients demonstrated a 6% rate of upstaging in patients with stage IIb disease using pathologic staging.^16^ Other studies have also noted a higher rate of upstaging in patients with axillary disease and recommended that metastatic workup should not be performed in a patient with a clinically negative axilla.^16,17^

We propose several reasons for our overall higher rate of upstaging. First, our analysis included all patients with invasive breast cancer. This included those who were symptomatic for metastatic disease, which may have contributed to a higher rate of upstaging. Second, a majority of our patients received a CT scan as part of their metastatic workup, which is more sensitive than the staging modalities (chest x-ray and ultrasound liver scan) used in some of the previously reported studies.

Finally, we were able to correlate clinical stage with pathologic stage in the subset of patients who received upfront surgery (Fig 2). There was a trend toward underestimating the clinical stage (cTN) at diagnosis when compared with the pathologic stage (pTN) despite taking the largest value of the three modalities (ie, clinical examination, mammogram, and ultrasound) as the cT and cN. This underestimation using clinical parameters appeared to be most pronounced in patients with pathologic stage IIB, where 60% were underestimated as having clinical stages I and IIA, and in pathologic stage III, where 77% were clinically underestimated as having clinical stages I, IIA, or IIB. This may partially explain our higher rates of upstaging to stage IV in clinical stages IIB and III than that reported in the literature.

Boutros et al^18^ conducted a study of 2,059 patients and developed a nomogram predictive of distant metastasis in newly diagnosed patients with breast cancer. This nomogram was also validated using two other cohorts in the same study. They reported tumor size and nodal status as the only significant predictors of distant metastasis, which is similar to our findings. Our study, as well as the one by Boutros et al,^18^ showed that biologically aggressive variants of breast cancer, such as grade III, HER2-enriched (or triple-negative tumors) were not predictive of metastasis at initial presentation.

It has been previously reported that treating physicians and surgeons are reluctant to follow guidelines pertaining to screening for distant metastasis.^11,12,19^ There is a greater burden of advanced disease in our population compared with studies reporting data from the US National Cancer database.^14^ In this study, almost all patients were screened for metastatic disease by imaging studies. In our experience, this deviation from guidelines is at least partly due to the desire of self-paying patients to obtain a full metastatic workup, even in early disease.^20^ As demonstrated by previous studies, our study also showed low rates of upstaging to stage IV in clinical stages I (n = 0 of 40, 0%) and stage IIA (n = 6 of 107, 5.6%).

Our results echo the importance of avoiding routine metastatic workup in these subsets of low-risk patients because it leads to unnecessary cost, delay in treatment, and anxiety, especially among those with false-positive results who then require additional testing, such as an MRI or ultrasound scan. It has been shown that appropriate educational interventions can change surgeon attitudes and practices toward metastatic screening.^21^ In addition, our results also show approximately 50% concordance between clinical and pathologic stages, with a propensity for the clinical underestimation of TNM staging. Thus, some patients thought to have early-stage breast cancer using all available clinical data, such as examination and breast imaging, may actually have more advanced disease on pathologic examination. Because our upstaging to M1 in early breast cancer, especially in stage IIB, is high, obtaining metastatic workup in these patients may be useful.

There are some limitations to our study. The retrospective collection of data was challenging because of a lack of standardized clinical documentation (no standardized electronic medical record) and thus variation in documentation between breast consultants. This also prevented us from differentiating between patients who presented with symptoms and those who were asymptomatic for metastatic disease. Additionally, because of challenges with handling, retrieving, and reviewing paper charts, we were only able to include a small sample size of 370 patients.

In conclusion, our findings confirm guidelines that patients with node-negative early-stage invasive breast cancer should not routinely undergo a distant metastatic workup. Although receptor status in the current era is an important determinant of aggressiveness of disease, it should not be used to determine the need for metastatic workup. We recommend consideration for obtaining metastatic workup in patients clinically determined to have stage IIB disease or higher. Our findings also demonstrate a need for both patient and provider education to avoid unnecessary testing.
